# Correction: Characteristics of hospitalized patients during a large waterborne outbreak of *Campylobacter jejuni* in Norway

**DOI:** 10.1371/journal.pone.0259407

**Published:** 2021-10-27

**Authors:** Nicolay Mortensen, Solveig Aalstad Jonasson, Ingrid Viola Lavesson, Knut Erik Emberland, Sverre Litleskare, Knut-Arne Wensaas, Guri Rortveit, Nina Langeland, Kurt Hanevik

The 8^th^ author is missing an additional affiliation. Nina Langeland is also affiliated with #8: Department of Medicine, Norwegian National Advisory Unit on Tropical Infectious Diseases, Haukeland University Hospital, Bergen, Norway.
